# Expressing Personality Through Non-verbal Behaviour in Real-Time Interaction

**DOI:** 10.3389/fpsyg.2021.660895

**Published:** 2021-11-26

**Authors:** Maryam Saberi, Steve DiPaola, Ulysses Bernardet

**Affiliations:** ^1^School of Interactive Arts and Technology, Simon Fraser University, Vancouver, BC, Canada; ^2^Aston Institute of Urban Technology and the Environment (ASTUTE), Aston University, Birmingham, United Kingdom

**Keywords:** personality, non-verbal behaviour, behaviour regulation, virtual human, synthetic psychology

## Abstract

The attribution of traits plays an important role as a heuristic for how we interact with others. Many psychological models of personality are analytical in that they derive a classification from reported or hypothesised behaviour. In the work presented here, we follow the opposite approach: Our personality model generates behaviour that leads an observer to attribute personality characteristics to the actor. Concretely, the model controls all relevant aspects of non-verbal behaviour such as gaze, facial expression, gesture, and posture. The model, embodied in a virtual human, affords to realistically interact with participants in real-time. Conceptually, our model focuses on the two dimensions of extra/introversion and stability/neuroticism. In the model, personality parameters influence both, the internal affective state as well as the characteristic of the behaviour execution. Importantly, the parameters of the model are based on empirical findings in the behavioural sciences. To evaluate our model, we conducted two types of studies. Firstly, passive experiments where participants rated videos showing variants of behaviour driven by different personality parameter configurations. Secondly, presential experiments where participants interacted with the virtual human, playing rounds of the Rock-Paper-Scissors game. Our results show that the model is effective in conveying the impression of the personality of a virtual character to users. Embodying the model in an artificial social agent capable of real-time interactive behaviour is the only way to move from an analytical to a generative approach to understanding personality, and we believe that this methodology raises a host of novel research questions in the field of personality theory.

## 1. Introduction

Humans show consistent patterns of thoughts, feelings and behaviour that distinguish one person from another and persist over time and situations (Higgins and Scholer, 2008). These observable patterns are what we refer to as “personality.” Personality can be construed from the angle of the mental model an observer infers based on the behavioural pattern or from the perspective of individual differences in regulatory dynamics of emotion and cognition. For example, individuals who are more extravert smile often, show more body movements and facial activity and exhibit more frequent hand and head movements (Borkenau and Liebler, 1992; La France et al., 2004). That the perception of personality is based on exhibited behaviour and that tendencies to engage in certain behaviours is due to personality traits is a duality that is also reflected by the two broad classes of personality theories and models. Factorial personality theories are a data-driven approach that is based on the analysis of a large pool of self and peer report items. The most widely used factorial theory is the “Big Five” model that distinguishes the five personality traits of Openness to experience (inventive/curious vs. consistent/cautious), Conscientiousness (efficient/organised vs. easy-going/careless), Extraversion (outgoing/energetic vs. solitary/reserved), Agreeableness (friendly/compassionate vs. cold/unkind), and Neuroticism (sensitive/nervous vs. secure/confident) (McCrae and John, 1992). The model has widespread acceptance in psychology, and its validity has been shown in numerous studies. Conversely, mechanistic models are theory-driven and based on hypothesised etiological processes. These models assume characteristic individual differences in emotional, motivational, and cognitive processes that lead to different stable personalities. The “BIS/BAS” and “CAPS” models are prominent examples of these types of models. The BIS/BAS model proposes that people differ in the sensitivity of their Behavioural Inhibition System (BIS, responsible for anxiety) or their Behavioural Approach System (BAS, responsible for impulsivity) (Gray, 1987). People with BIS tend to be more sensitive to moving away from unpleasant events and punishments, while those with BAS are sensitive to signals of reward and desired events. The Cognitive-Affective Processing System (CAPS) theory of personality explains individual differences as a “characteristic pattern of cognitions and affects [that] becomes activated through [a] distinctive network of connections”. (Mischel and Shoda, 1998). With the aim to bridge between factorial and mechanistic approaches, Read et al. (2010) proposed a neural network model of structure and dynamics of personality based on neurobiological findings. Some mechanistic models have a computational realisation (e.g., Karimi and Kangavari, 2012; Read et al., 2018), however, they seldom make predictions about specific behavioural outputs.

In this manuscript, we present the “RealAct” (Saberi et al., 2015) model of personality-based behaviour regulation with the focus on the empirical evaluation of the model in real-time interaction between a simulated “Virtual Human” and a participant. In its current form, the model is purely non-verbal, meaning that it does not produce any verbal output or take any verbal input into account.

The specific, and novel, scenario for the interaction is the playing of several rounds of the Rock-Paper-Scissors games between the two parties. Methodologically, this means that we test the model by embodying it in a virtual human and controlling the behaviour of the agent. The primary hypothesis formulated by the “RealAct” model is that at the mechanistic level the two dimensions of Extraversion and Neuroticism can be explained by differences in affective processing, and by the mode and probability of the execution of non-verbal behaviour. Our secondary hypothesis is that these mechanisms lead to an attribution of different personality categories by an observer. The motivation behind the development of the RealAct is twofold. On the one hand, the model proposes the scientific hypothesis outlined above. On the other hand, being able to “equip” a virtual agent with different dimensions of personality, allows making them more believable and adaptive to the human they are interacting with.

### 1.1. Related Work

Many models that generate behavioural outputs come in the form of control architectures for Virtual Humans, i.e., computer-generated humanoid agents. André et al. (2000) developed computational models of emotions and personality for children's virtual puppet theatres, virtual sales presentations, and virtual guides for internet websites to make the interaction more enjoyable and closer to communication styles in human-human conversations. PERSEED is an architecture that was developed using a socio-cognitive perspective to build a model of personality for 3D virtual characters, with a focus on how the situation may affect any personality behaviour exhibited (Faur et al., 2013). No experiment has been performed, however, to reveal the application of this model in the social and situation-based interaction. Neff et al. (2011), limited their study to investigate the correlation between FFM's neuroticism trait and changes in conversations and nonverbal behaviour. They found that the presence of self-adaptors (movements that often involve self-touch, such as scratching) made characters look more neurotic. ALMA (A Layered Model of Affect) (Gebhard, 2005) is designed to provide a personality profile with real-time emotions and moods for 3D virtual characters. The emotions and moods are computed based on the appraisal of relevant inputs. The concentration in this study is on modulating the appraisal process, but there is no mapping between nonverbal behaviour and personality traits. Kshirsagar (2002) devised a personality model of emotional 3D virtual characters that used Bayesian Belief Networks and a layered approach for modelling personality, moods, and emotions. The focus of this work was only on emotional personality. Similarly, Su et al. (2007) designed a system to control affective storey characters with parameters for personality and emotion. They developed a hierarchical fuzzy rule-based system to control the body language of a storey character with personality and affect. In this system, storey designers specify a storey context with personality and emotion values with which to drive the movements of the storey characters. Poznanski and Thagard (2005) developed a neural network model of personality and personality change: SPOT (Simulating Personality over Time). Personality-based predispositions for behaviour, moods/emotions, and environmental situations specify the output behaviour. In their model, personality develops over time, which is, in turn, based on the situations encountered.

While some of the architectures described above attempt to formulate a psychological hypothesis, many of them lack a grounding in mechanistic explanations and are of a statistical nature. A notable exception are Shvo et al. (2019) who propose a comprehensive model that focuses on the integration of personality with emotion, motivation and behaviour planning. In their model, personality modulates the intensity of experienced emotions as well as the importance a person assigns to different motivations. They e.g., “correlate extraversion with social contact, power, and status.” The modulation affective state by personality factors is similar to our model, however, it is not clear to what extend the model by Shvo et al. (2019) would afford the control of real-time interaction and non-verbal behaviour generation. Recently, Sajjadi et al. (2019) have proposed an emotion model that integrates personality at its core. The model supports voice-based turn-taking interaction between a virtual human and a user. In its current form, the model only integrates conversation options that cause negative changes in the affective state of the agent. The emotion system is based on the Pleasure-Arousal-Dominance model of affect (Mehrabian and Russell, 1974) and integrates a single personality dimension of extraversion–introversion. The model controls speech, lip-syncing, eye gazing, facial expressions, postures, and gestures. However, the authors do not provide a detailed quantitative mapping of personality dimensions to non-verbal behaviour. Ishii et al. (2020) have taken a machine learning-based approach to investigate the link between personality and non-verbal behaviour. Based on an annotated corpus, they have trained a model to generate non-verbal behaviour associated with speech. Interestingly, they have found the dimension of extraversion to be less useful in improving the prediction models for non-verbal behaviours.

### 1.2. Virtual Humans

Virtual Humans are a form of human-machine interface where the machine is communicating with the user through the representation of an artificially intelligent animated human form. Broadly speaking there are two complementary ways in with virtual humans are used; Firstly, as stand-ins for biological humans, in what can be described as “social interaction as a service,” and secondly, as a tool to develop and test models of human cognition, affect, personality, and behaviour regulation. In the prior application case, virtual humans are developed e.g., as virtual therapists (Ranjbartabar et al., 2019), for guided relaxation (Tunney et al., 2016; Dar et al., 2019), in language teaching (Scassellati et al., 2018), various forms of training such as communication and social skills (Razavi et al., 2016; Tanaka et al., 2017), negotiation skills (Broekens et al., 2012), and public speaking (Batrinca et al., 2013). For such virtual humans to create an efficient and satisfactory user experience, they have to behave coherently and consistently throughout the interaction (Faur et al., 2013; Saberi, 2016b). Conversely, a lack of consistency in behaviour is assumed to have a negative impact on training and learning efficiency (Van den Bosch et al., 2012). Indeed, Mcrorie et al. (2012) showed that to be convincing, virtual humans need “a coherent set of behavioural responses that can be interpreted by a human observer as indicative of a personality.” Participants prefer to interact with characters that show consistent behaviour (Isbister and Nass, 2000) and incorporate non-verbal behaviour-based personality increases the users' perceived sense of social presence (Sajjadi et al., 2019).

#### 1.2.1. Virtual Human for Testing Psychological Models

Modelling in psychology faces the challenge that on the one hand purely theoretical models tend to be unspecified and inconsistent (Mischel and Shoda, 1998), while on the other hand, large-scale models are inherently difficult to test (Heckhausen and Heckhausen, 2008). In the presented work we tackle these challenges by embedding the models in a real-world context of action execution that focuses on the functional aspect and the generation of overt behaviour. Concretely, we “embody” our model in an autonomous computer-generated human that can interact with biological human participants. A key requirement for using virtual humans in this way is that there is a qualitative equivalence in how they are perceived. In the domain of embodied conversational agents (ECA) Cassell and Tartaro (2007) have introduced the concept of “intersubjectivity” that asks if, in explicit and implicit communication (Dar and Bernardet, 2020), the human user reacts to an agent in the same way they react to other humans. expressions of emotions in virtual humans can be perceived similarly to human emotions. With regards to the relationship between personality and non-verbal behaviour, studies have shown that consistent behavioural patterns of virtual humans are interpreted by users as a distinct personality (Mcrorie et al., 2012). Conducting a nuanced analysis of personality impression using standardised, validated personality questionnaires Castillo et al. (2018) were able to show that virtual humans can convey personality through appearance and behaviour and that people do treat these agents as though they have human-like personalities. Importantly, each physical channel (visual and acoustic) conveys information about personality, that personality is multi-modal and “any attempt to study or design personality by solely focusing on voice or face characteristics will most likely fail.”

One potential limitation of using virtual humans form model embedding, comes in the form of the “Uncanny Valley” Hypothesis (UVH), originally formulated by the roboticist Mori (1970) that predicts a specific, non-linear relationship between human-likeness and likeability of an artificial agent. The “Uncanny Valley” Hypothesis posits that initially, likeness and likeability show a linear relationship; the more the agent looks like a human, the more it is liked. However, when the similarity exceeds a certain, relatively high level, the relationship inverts, and the agent is perceived as uncanny. However, after this valley, the relationship becomes positive again. This dip in likeability can be an issue when using virtual humans as a tool to embed models as users might be made feel uncomfortable by the presence of the agent. Studying computer-generated faces, MacDorman et al. (2009) have shown that the faces do not necessarily become more “eerie”, the more photo-realistic they are. However, distortions and aberrations from the norm (e.g., enlarged eyes) lead to more eeriness, the more photo-realistic the face is. emotional expressivity, is limited in the upper face. Their results indicate the level of uncanniness is dependent on what emotion is being communicated. However, Kätsyri et al. (2019) observed a linear, positive relationship between human-likeness and affinity; in their study, the least human-like faces elicited the most aversive response. In the same vein, a similar relationship was found by Seymour et al. (2019). In their study, increased photo-realism was positively related to trustworthiness and affinity. In conclusion, despite its appeal and popularity, the relationship between human-likeness and likeability as postulated by the “Uncanny Valley” hypothesis is not clearly established, not least due to the challenges associated with operationalising human-likeness (Ho and MacDorman, 2016). Especially in the domain of Virtual Humans, research shows a nuanced picture. Some of the findings, e.g., that aberrations from the norm in highly realistic faces lead to a higher degree of eeriness, actually supports the argument of using virtual humans as a tool to embed models, as the level of eeriness can directly be used as an indicator of the realism of the behaviour generated by the model.

## 2. The “RealAct” Model of Personality-modulated Behaviour Regulation

**Figure 1:**
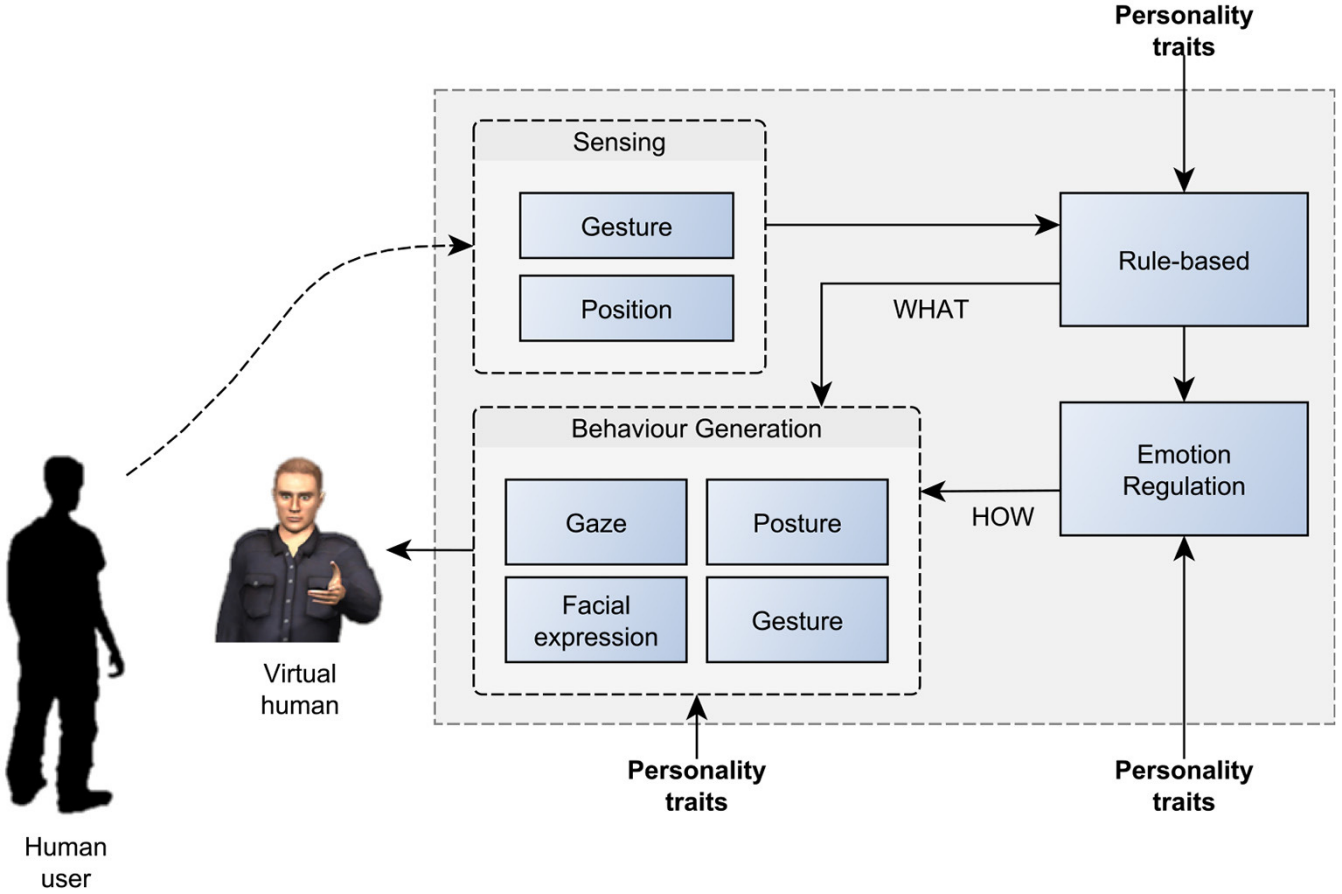
The system architecture and how personality impacts its different modules. Personality traits affect the production of gestures, posture, gaze and facial expression.

Our model is based on systems theoretical principles that have a long tradition in modelling animal as well as human behaviour, e.g., to understand basic motivational systems (Toates and Archer, 1978) and attachment dynamics, respectively (Bischof, 1975). Especially, control theoretical concepts have become pervasive in psychology and biology, e.g., in the form of the Perceptual Control Theory (Bell and Pellis, 2011), Dynamic Process Theory (Vancouver, 2008), and Motivational Control Theory (Hyland, 1988). The model is a hybrid architecture that consists of a continuous component for Emotion Regulation and an event-based component for Rules-based Behaviour (Figure 1). The continuous component maintains the internal affective state and integrates external events. This component is also the main driver of emotion expressions. Concurrently, the rule-based system is responsible for the logic and flow of the interaction with the user and contains additional behaviour controllers and a behaviour scheduler. In conjunction, these two components provide the system to control gestures, postures, gaze and facial expression. In our model personality deferentially affects both, the internal regulation of emotion and behaviour execution. As the scope of the model, we define the real-time interaction between a virtual human and a participant. The specific scenario for the interaction is the playing of several rounds of the rock-paper-scissors games between the two parties. This means that the model has to support the generation of real-time non-verbal behaviour based on the integration of open-ended environmental and social input. To maintain the interaction, the model needs to sustain consistent and believable behaviour of a realistic 3D agent over an extended period of time. So far, very few models have been proposed that are not only mechanistic and psychologically plausible, but also have the ability to produce actual behaviour based on non-verbal input. Moreover, our model proposes a quantified and evidence-based relationship between personality and behaviour selection and execution parameters (Tables 1–**4**).

**Table 1:** Based on empirical findings, five dimensions for the effect of personality parameters on the valence and arousal are defined (for emotional valence, initially experienced value of emotion, frequency of chance of valence and reaction to stimuli; and for emotional arousal, initial value and arousal change in response to positive and negative stimuli).

	**Valence**		**Arousal**		
	**Initial value**	**Reaction to stimuli**	**Change freq**	**Initial value**	**Reaction to stimuli**
HELS	0 (γ)	High to positive & negative (α)	High (β)	Positive (β)	High (β)
HEHS	Positive (γ)	High to positive (α)	Low (β)	Negative (β)	Low (β)
LELS	Negative (γ)	High to negative (α)	High (β)	Positive (β)	High (β)
LEHS	0 (γ)	Low to positive & negative (α)	Low (β)	Negative (β)	Low (β)
Neutral	0 (γ)	Normal to positive & negative (α)	Low (β)	0 (β)	Normal (β)

*α: Carver and White (1994), β: Funder and Sneed (1993), γ: Larsen and Ketelaar (1991)*.

### 2.1. Model Inputs

The sensing module receives input from real-time sensors and provides the model with information about the human user's hand gestures and position in space. The outputs of the system are behaviour commands sent to the animation engine for three modalities: facial expressions, postures/gestures and gaze movements. These commands are dynamically fed to the animation engine, and performed by the virtual agent during the simulation. Behaviour controllers generate commands for controlling the facial expressions, postures/gestures, and gaze of the agent. The behaviour scheduler prioritises and selects an action from multiple behaviour requests generated by behaviour controllers and sends the one with the highest priority to the animation engine.

### 2.2. Role of Personality Parameters

Our model includes personality-related mechanisms at two levels: Firstly, in the way emotions are integrated and expressed, and, secondly, in the way, behaviours are executed (Figure 1). The dimensions of Extraversion and Emotional Stability are well established and proposed to have a biological basis (Ostendorf and Angleitner, 1994). Importantly, there is empirical evidence regarding the link between them and nonverbal behaviour (Campbell and Rushton, 1978; Borkenau and Liebler, 1992). We focus on the combination of two traits—Extraversion and Emotional Stability. This allows defining four personality categories: High Extraversion/Low Stability (HELS), High Extraversion/High Stability (HEHS), Low Extraversion/Low Stability (LELS) and Low Extraversion/High Stability (LEHS).

### 2.3. Rule-Based Behaviour

Because of the discrete and turn-based nature of face to face interaction, the component for the rule-based control is implemented as a finite state machine (FSM). Interaction configurations and rules are set at the beginning of the interaction and determine the rules specific to the scenario of the interaction e.g., to which location in the environment the agent wants to direct the user. A state machine is a set of input events, output events, and states. The FSM is in one state at a time and changes from one state to another when an event or condition is triggered. Hence, an FSM is defined by a list of states and triggering conditions for each transition. Firstly, our model's FSM controls the turn-taking behaviour of the interaction, i.e., based on the interaction scenario, the turn-taking behaviour can be synchronised using users' actions or environmental inputs. Secondly, the FSM determines what agent's gestures and gaze behaviour is corresponding to the events, conditions, goals and strategies. An example of the working of the FSM is turn-taking between the agent and the user that can be synchronised using the coordination of the user in the space; the agent will only act if the user is standing still and not moving. In the FSM, specific events or information triggers corresponding gestures from the agent. A second example is the guidance of the user to a specific spatial location. The agent points to the location and gazes at the user to encourage them. Based on the predefined goals for the scenario of the interaction, and inputs the agent gets from the environment, they decide what state they should transfer to and what gestures they need to express in response to the user.

### 2.4. Emotion Regulation

The emotion regulation system in our model is based on the “Circumplex” model of affect proposed by Russell (1980). In this model, each emotion is characterised by a linear combination of Valence (pleasure–displeasure) and Arousal (arousing–not arousing). Some versions of this model include a third dimension of Dominance (Mehrabian and Russell, 1974), however, this dimension has received much less attention (Bakker et al., 2014). Some current emotion models for virtual humans make use of this third dimension e.g., Boulic et al. (2017). Becker-Asano (2014) also introduce the notion of “mood” as a more persistent affective state that has a modulatory effect on valence and arousal. In our model, emotions are updated continuously. Effectively, the current emotional valence and arousal are dependent on how long the agent has been in a specific state. Three kinds of triggers elicit emotional valence and arousal responses: events during the interaction between the agent and the environment, events related to the rock-paper-scissors game played in the interaction, and internal events.

### 2.5. Valence and Arousal Triggers

Several psychological studies indicate that emotion is contagious (Lundqvist and Dimberg, 1995). Thus, the positive valence of the agent increases if they sense a user's positive emotion (Lundqvist and Dimberg, 1995). A signal of potential gains increases valence while the signal of potential losses decreases valence (Wilson and Kerr, 1999). In the rock-paper-scissors scenario, we assume valence is increasing as the agent wins and decreases as they lose in each round. Thus, on the one hand, positive feedback from the user such as waving and smiling, as well as positive game events such as winning in the rock-paper-scissors game, increase the valence. On the other hand, negative feedback from the user and interaction scenario decrease the generated valence. As for valence, the generated arousal is a linear function of user and interaction scenario feedback. Uncertain cues, competition, challenges, reward and punishment typically increase arousal (Waxer, 1977; Napieralski et al., 1995). In addition, increasing the difficulty of the game leads to higher arousal (Waxer, 1977; Bartneck, 2002). Since arousal is in direct relationship with the difficulty of the game, in the rock-paper-scissors game scenario, we assume the agent's excitement increases as it gets closer to the “Go” state and decreases as it gets to the “Wait” state. Since psychological data shows that repeating the game again and again decreases the experienced arousal (Conati, 2002), during the rock-paper-scissors game the repetition of the game cycles have a negative effect on the agent's emotional arousal. While the model does not include a distance regulation component *per se*, a user invading the personal space of the agent will trigger an increase in the arousal of the interaction.

#### 2.5.1. Trigger Weights

**Figure 2:**
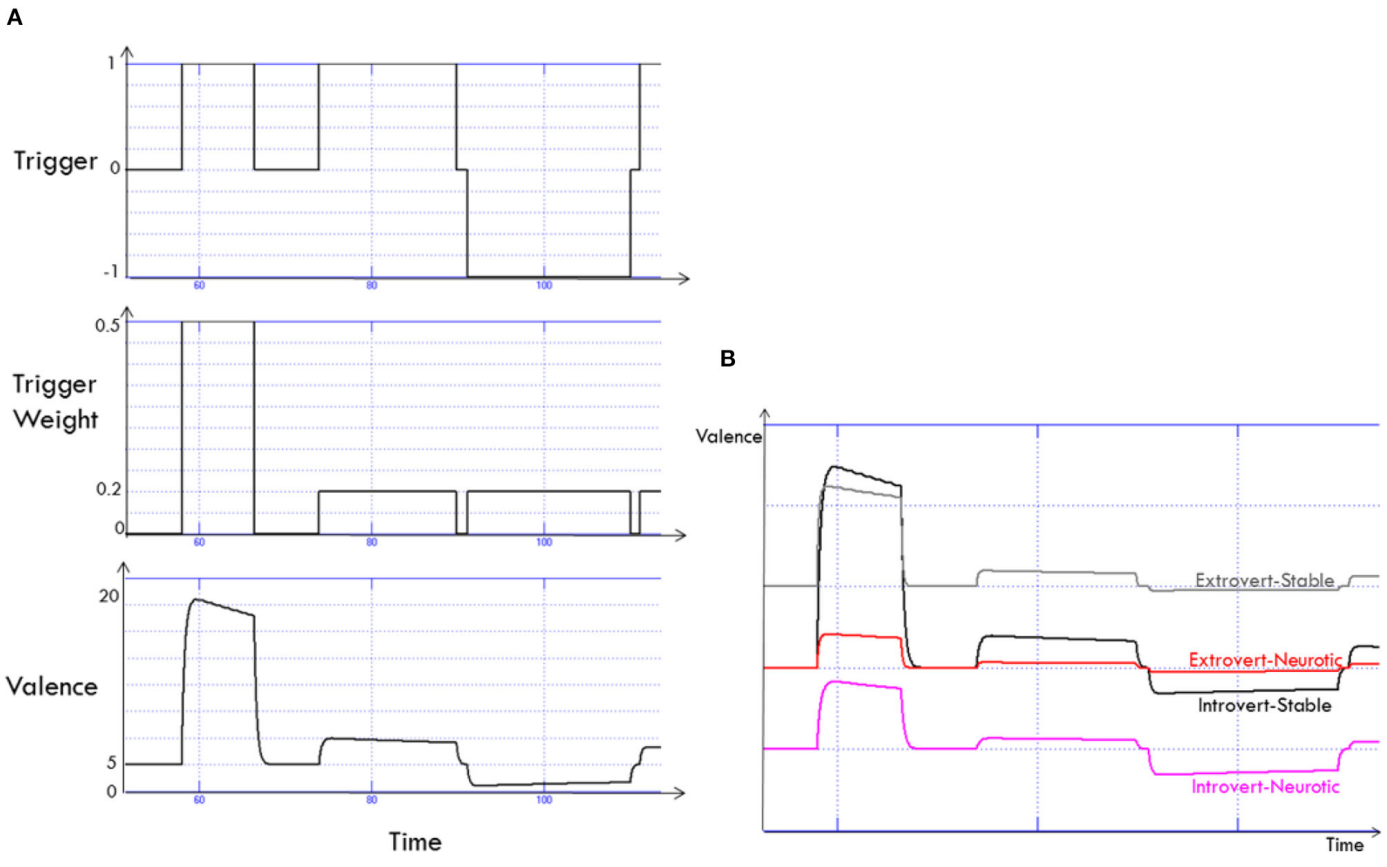
The generation of valence over time is a function of triggers that have different weights **(A)**. Comparison between dynamics of the Valence for four different personality categories **(B)**.

The level of impact of a trigger on valence and arousal is a function of how important the trigger is in satisfying the agent's needs. Based on Maslow's “hierarchy of needs,” we define three categories of input triggers: Self-esteem, love/belonging and safety (Maslow, 1970). Triggers related to safety have more impact on arousal and valence than those related to love/belonging, and, in turn, have higher importance than those related to self-esteem. A user invading the personal space of the agent jeopardises the need for safety and has the highest impact on arousal. Conversely, smiling back at the user corresponds to the need for being loved, which has lower importance. Trigger weights are used to differentiate between the effects of different inputs. Figure 2A) shows an example of the time course of input triggers, the weight of each trigger and the resulting valence change.

#### 2.5.2. Personality Factors

The core hypothesis of our model is that the emotional reaction to positive and negative feedback is a function of extraversion and stability. The quantitative differences between the four personality categories, implemented in our model, in terms of generation of valence and arousal are listed in Table 1. For example, high extravert-high stable (HEHS) individuals are more responsive to positive stimuli (Carver and White, 1994), and experience more positive emotions in general (Larsen and Ketelaar, 1991). Since more extravert individuals tend to show stronger emotional responses to the positive feedback (for example, winning in a game), the valence curve has a high exponential rise. However, individuals who score high in extraversion are not typically sensitive to negative feedback; hence when feedback is negative, the valence decreases more slowly. Individuals scoring low on stability, on the other hand, typically show a stronger response to negative than positive stimuli (Carver and White, 1994). Therefore, valence decreases with a higher rate of response to negative feedback, and such individuals experience more negative emotions in general (Funder and Sneed, 1993). Figure 2B shows the emotional valences generated by the model's emotion regulation module for four personality categories.

#### 2.5.3. Valence and Arousal Attractors

**Figure 3:**
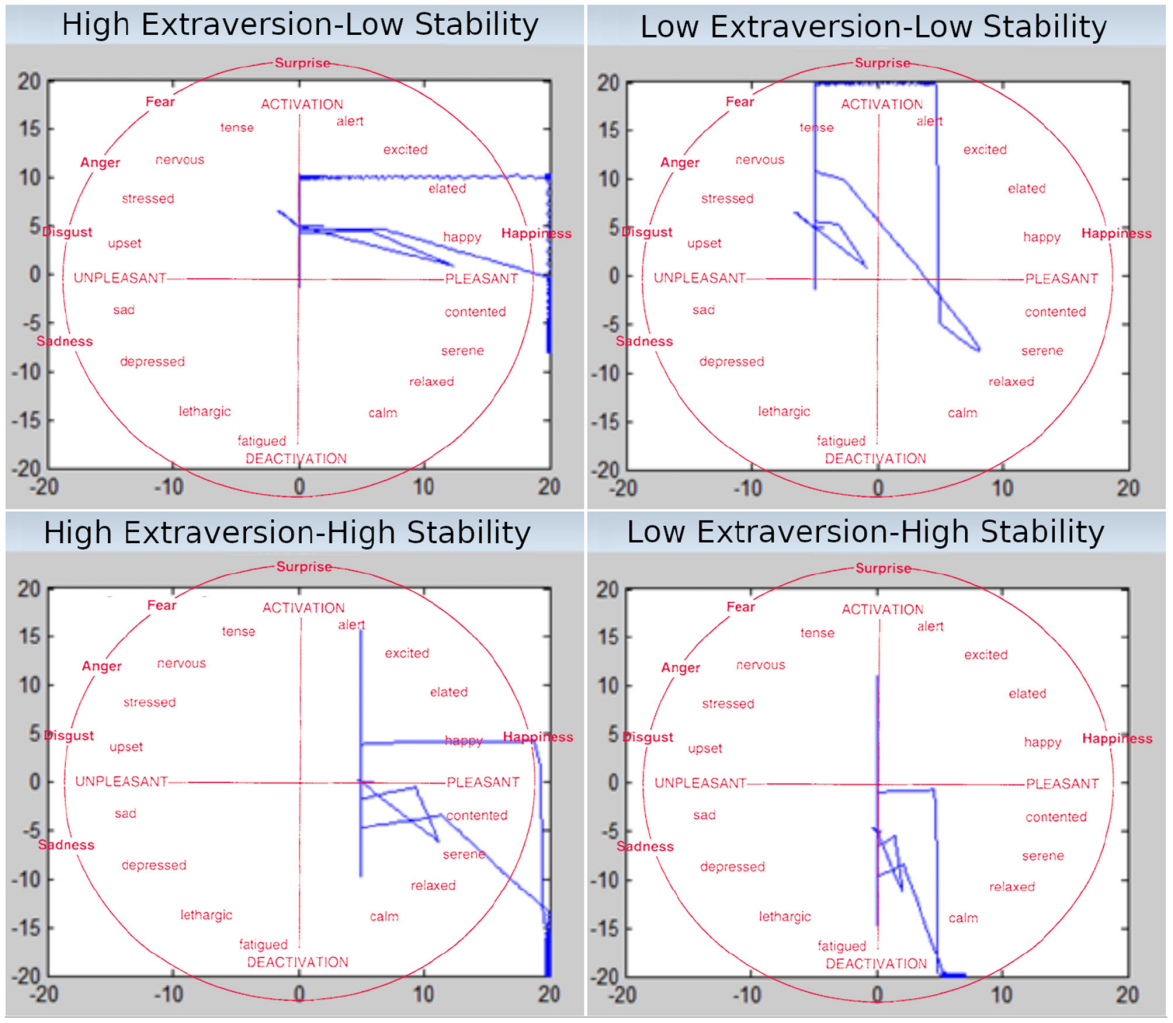
Effect of the personality parameters on the time course of Valence and Arousal. The x dimension denotes Valence, the y dimension Arousal. The red overlaid text show the circumplex model of affect (Russell, 1980).

In our model, valence and arousal constitute a 2-dimensional space. Over time, the different personality categories will yield different “attractors” in this space Figure 3. The preference of a certain location within the space generated by our model can be compared with theoretical predictions. Indeed, our model generates valence and arousal attractors that are comparable to Heller's model (Schmidtke and Heller, 2004). For instance, high extravert-high stable (HEHS) generates mostly positive valence and a combination of negative and positive arousal.

### 2.6. Behaviour Controllers Modules

The model controls four aspects of behaviour: Facial expression, posture, gesture and gaze. For each of the domains, three kinds of behaviour are defined: idle, reactive and communicative. The idle behaviour is a dynamic set of behaviour sequences consisting of subtle movements of various body parts such as gaze, head and hand movements. Idle behaviour is based on the observation that humans tend to keep moving their body even if they are not engaged in a specific task, i.e., they shift their body weight, scratch themselves, or move their head around. Reactive behaviour is in response to a sudden or unexpected change of environment and an automatic behaviour. For instance, if the user gets too close, the agent automatically moves back and adjust their personal space (Bernardet and DiPaola, 2015). The reactive behaviour has the highest priority. The communicative behaviour is triggered in response to interaction goals. For example, in the Rock-Paper-Scissors game (RPS) interaction, the agent shows the rock hand gesture. Communicative behaviour has a higher priority than idle behaviour and will replace it if they are triggered at the same time.

#### 2.6.1. Facial Expression Controller

Triggered by external and internal stimuli, changes are continuously generated in the emotion regulation module. These emotions then are expressed through facial behaviour. For the emotions that are revealed externally through our agent's face to the user, generated values of valence and arousal are mapped to Ekman's Action Coding System to generate facial expressions (Ekman and Friesen, 1974). To map the valence and arousal and facial action units, we use the data from a study by Boukricha et al. (2009), that applied a “reverse-engineering” method to determine how facial action units map to the Pleasure-Arousal-Dominance model. Based on Boukricha et al. (2009)'s data, the intended valence and intended arousal were driving the facial expression at five levels (low, medium-low, medium, medium-high, and high). The dominance dimension of the PAD model of affect was not taken into account in the control of the facial expression. Personality affects the expression of emotion in three ways: Intensity of emotion expressed, how much emotion is filtered out and if the agent shows facial twitching (Table 2). For instance, more extravert individuals tend to express their emotions more freely and do less filtering (Gill and Oberlander, 2002). Conversely, low stability correlates with the presence of involuntary face twitches and head jerkiness (Waxer, 1977).

**Table 2:** Based on the personality category of the agent, the amount for activated action units is adjusted.

	**Action_Unit_Amount**	**Philtre threshold**	**Face_Twitches**
HELS	High (γα)	Low (β)	Yes (γ)
HEHS	High (γα)	Low (β)	No (γ)
LELS	Low (γα)	High (β)	Yes (γ)
LEHS	Low (γα)	High (β)	No (γ)
Neutral	Normal (δ)	Normal (β)	No (γ)

*In addition, based on personality category, if the amount assigned to an action unit is less than a threshold it will be filtered out (considered as internal and not expressed emotional states). α: Funder and Sneed (1993), β: Gill and Oberlander (2002), γ: Waxer (1977), δ: Boukricha et al. (2009)*.

#### 2.6.2. Gaze Controller

**Figure 4:**
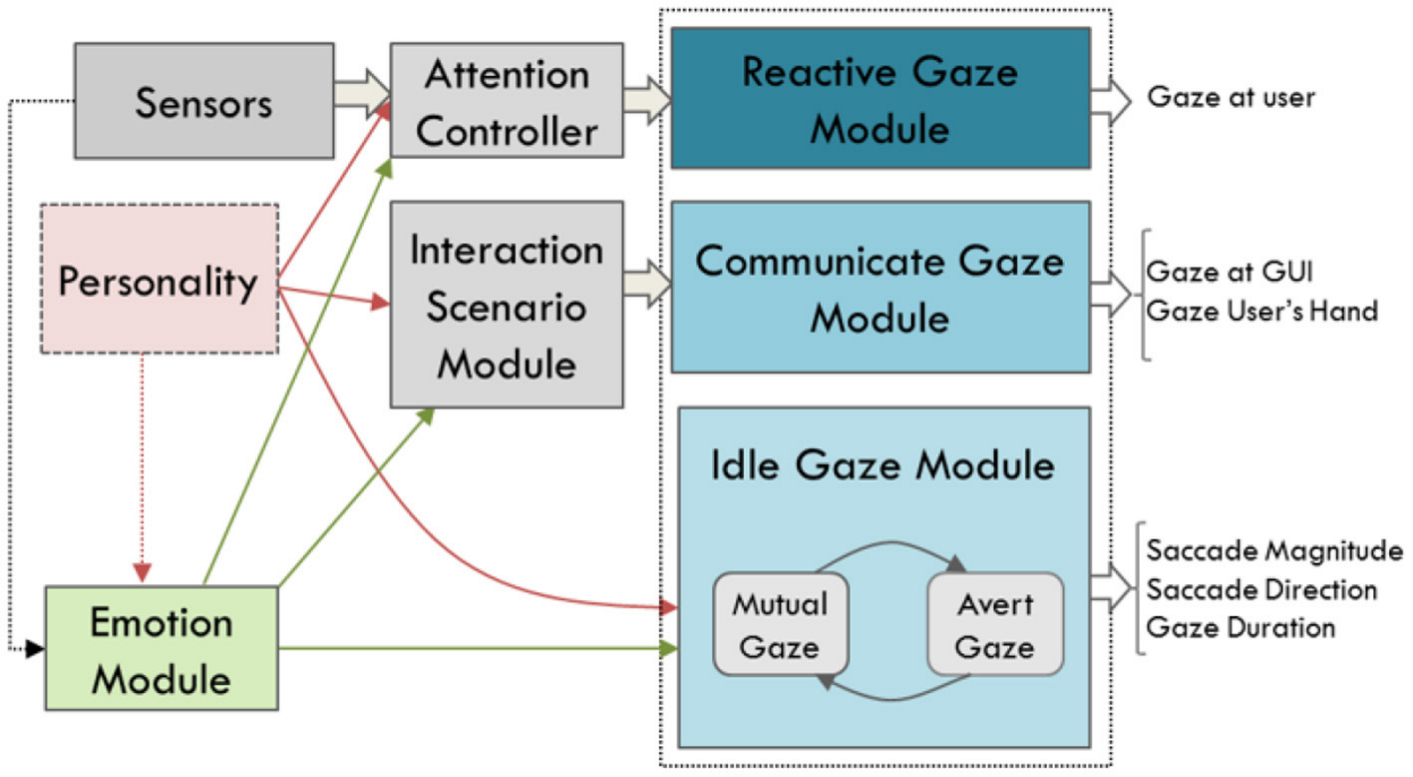
Structure of the Gaze Control module. Reactive gaze has a high level in the hierarchy and is responsive to environment changes. Communicative gaze is responsive to the agent's goal and scenario of the interaction. Idle gaze has a low level of importance, and other gaze behaviour will overwrite them.

Gaze behaviour is a combination of movements of the agent's eyes, head, chest, back and torso. Additionally, it includes blinking frequency. Our model contains three categories of gaze behaviour: Idle, reactive and communicative (Figure 4). The idle category is employed for the gaze behaviour when the agent is not engaged in a specific behaviour. The kinematics of the idle gaze module is based on the “Eyes Alive” gaze model proposed by Lee and colleagues (Lee et al., 2002). Their eye behaviour model is derived from statistical models of human eye-tracking data, and defines two gaze states: “mutual” and “avert.” In mutual gaze, the agent gazes at the user, while in the avert state the agent is looking away. The Eyes Alive model is based on three parameters saccade magnitude, saccade direction, saccade duration. Reactive gaze behaviour is driven by sensory input, such as the interaction partner's movements. Through this mechanism, the agent's gaze follows the user as they move into the space in front of the agent. The communicative category generates gaze behaviour that communicates meaning to the user or is triggered by a change in the state of the interaction. For instance, in a rock-paper-scissors game, the agent gazes at the graphic user interface (GUI) which has real-time updates of the game statistics. Eye movements are usually followed by a head rotation in the same direction. A saccade normally does not exceed 15 degrees (Bahill et al., 1975). Hence, if the generated amplitude is more than 15 degrees, a head movement in the same direction as the eyes is generated. When the head rotates to a new position, eye movement is automatically generated and layered on top of the head and torso movement.

The three categories of gaze behaviour—idle, reactive and communicative—are creating potentially conflicting behaviour execution requests. The role of the “Behaviour Scheduler Module” (Section 2.8) is to select the appropriate behaviour according to their priority.

Table 3 gives a detailed description of all the gaze parameters to generate different gaze behaviour for the different personality categories. The direction, duration, frequency of the gaze and speed of the head movements are controlled to give different impressions of personality. For instance, if the agent is low extravert-low stable, they avert their gaze more frequently (COOK and SMITH, 1975), but for short periods of time (Napieralski et al., 1995), mostly gazes down left and right (Tankard, 1970; Fukayama et al., 2002) and moves its head with a low speed (Borkenau and Liebler, 1992). For the blinking behaviour in our model system, we use statistical data from human blinking behaviour and how emotional state is affected by personality. Based on Itti and colleagues (Itti et al., 2004), people blink 11.6 times per minute. In higher arousal, people tend to blink more frequently (Kanfer, 1960). Thus, as with other parameters of the gaze, we defined a factor to change the blinking frequency during the simulation based on the personality category. Communicative gaze is triggered based on the scenario of interaction. Although these behavioural acts do not contribute to the expression of personality, they are necessary to have an active, realistic interaction. For instance, immediately after playing the hand in the rock-paper-scissors game, the agent looks at the hand of the user to see what they played, or looks at the GUI to see and confirms the result.

**Table 3:** Summary of empirical findings on the expression of personality traits through gaze behaviour.

	**Gaze type**	**Gaze dur**	**Gaze freq**	**Gaze dir**	**Blink freq**	**Head speed**
HELS	Both mutual & Avert (ηκ)	Long–Short (ιζ)	High–Low (λ)	U/C/L/R (γβαδ)	High (ϵ)	High (νξ)
HEHS	More mutual (ηκ)	Long–Med (ιζ)	High–Medλ)	U/C/L/R (γβαδ)	Low (ϵ)	High (νξ)
LELS	More avert (ηκ)	Short (ιζ)	Low (λ)	D/L/R (γβαδ)	High (ϵ)	Low (νξ)
LEHS	Both mutual & Avert (ηκ)	Short–Med (ιζ)	Low–Med (λ)	D/L/R/C (γβαδ)	Normal (ϵ)	Low (νξ)
Neutral	Both mutual & Avert (θ)	Normal (θ)	Normal (θ)	U/D/L/R/C (θ)	Normal (μ)	Normal (θ)

*Six dimensions of gaze behaviour are addressed: head speed, blinking frequency, type, direction, frequency and duration of gaze. U, D, C, L, R, respectively, stand for Up, Down, Centre, Left, and Right*.

*α: Rauthmann et al. (2012), β: Fukayama et al. (2002), γ: Tankard (1970), δ: Gill and Oberlander (2002), ϵ: Kanfer (1960), ζ: Lagomarsino et al. (1998), η: Larsen and Shackelford (1996), θ: Lee et al. (2002), ι: Napieralski et al. (1995), κ: Vandromme et al. (2011), λ: COOK and SMITH (1975), μ: Itti et al. (2004), ν: Simpson et al. (1993), ξ: Borkenau and Liebler (1992)*.

#### 2.6.3. Gesture and Postures Controller

**Figure 5:**
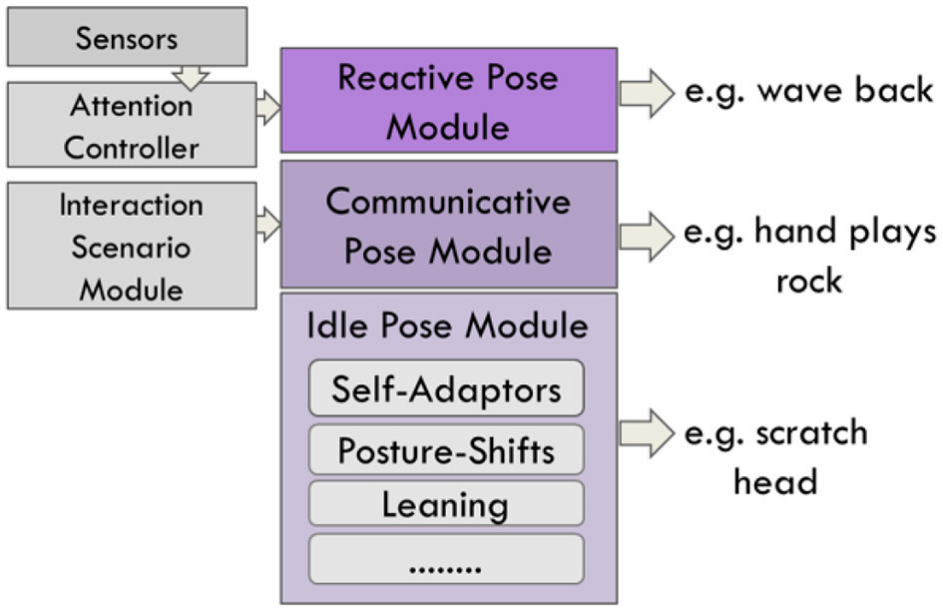
Structure of the gesture and post controller. Three types of gestures and pose behaviour are generated: Reactive, communicative and idle.

Similar to gaze behaviour, gestures can be reactive, communicative, or idle (Figure 5). Reactive gestures are responsive to the environment, such as the agent waving back at a user when that user waves at them. Communicative gestures and poses are generated based on the scenario of the interaction, such as the “rock” hand gesture in a rock-paper-scissors game. With these types of gestures, strong emotional reactions can be accompanied by a set of emblems. For example, if the agent is very angry, they position their hand into a fist while making a frowning facial expression. Emblems, illustrators and self-adaptors are proposed by Ekman and colleagues as three classes for hand movement behaviour (Ekman and Friesen, 1972). Additionally, we use self-adaptors such as “scratching the head” and “holding two hands together.” Same as for the idle gaze, idle poses and gestures are performed while the agent is not performing any specific task.

The chance of selecting each of the gestural/postural animations from the mentioned categories is a function of the simulation time, emotional state of the agent (valence-arousal) and personality parameters of the agent (Extraversion and Stability). These three parameters can specify if the pose is occurring if it is either fast or slow and how frequent the occurrences of a category of gestures are.

The model incorporates five dimensions for expressive idle gestures and posture: (1) Posture-shift behaviour, (2) Self-adaptor behaviour, (3) Leaning behaviour (lean forward, no lean and lean backward), (4) Twitches (true or false) and (5) Spaciousness of gestures (true or false). These five dimensions can have three different frequencies (high, mid, low) and three speeds (fast, mid, slow). We map these dimensions onto our personality categories (Table 4). For instance, high extravert-low stable individuals show idle poses and gestures more frequently and faster. High extravert individuals tend to lean back more and use more space when gesturing and posing (Schulman and Bickmore, 2012). Individuals scoring low on stability tend to twitch, and their poses and gestures are jerky (Campbell and Rushton, 1978). These five expressivity dimensions for gestures and poses were chosen because, firstly, these dimensions are important in creating the impression of personality, secondly, the dimensions have in general or in parts been used in several studies on affective computing and for creating the impression of personality and emotion, and, thirdly, it was feasible to synthesise these dimensions using my animation toolkit's provided features.

**Table 4:** Relationship between personality, and gesture and pose behaviour.

	**Gest/pose freq**	**Lean**	**Posture-shift freq**	**Posture-shift speed**	**Self-adaptor freq**	**Self-adaptor speed**	**Twitches**	**Spacial extend**
HELS	High (αβ)	Back (γϵ)	10% (*zeta*η)	High (ικ)	90% (λδ)	High (κη)	Yes (θ)	Yes (γβ)
HEHS	Low (αβ)	Fwd (γϵ)	90% (*zeta*η)	High (ικ)	10% (λδ)	High (κη)	No (θ)	Yes (γβ)
LELS	High (βα)	Back (γϵ)	10% (ζη)	Low (ικ)	90% (λδ)	Low (κη)	Yes (θ)	No (γβ)
LEHS	Low (βα)	Fwd (γϵ)	90% (ζη)	Low (ικ)	10% (λδ)	Low (κη)	Yes (θ)	No (γβ)
Neutral	Norm (αβ)	Norm (γϵ)	60% (*zeta*η)	Norm (ικ)	40% (λδ)	Norm (κη)	No (θ)	No (γβ)

*The following dimensions for gestures/postures are proposed: Frequency of gestures and postures in general; Leaning behaviour; Posture-shifts behaviour frequency and speed; self-adaptors behaviour frequency; and speed and whether twitching and spacious poses and gestures are present. In order to differentiate the four modelled personality categories, the above expressivity parameters are used to adjust the behaviour. α: Riggio and Friedman (1986), β: Neff et al. (2010), γ: Lippa (1998), δ: Campbell and Rushton (1978), ϵ: Frank (2007), ζSchulman and Bickmore (2012), η: Borkenau et al. (2004), θ: Waxer (1977), ι: Simpson et al. (1993), κ: Borkenau and Liebler (1992), λ: Neff et al. (2011)*.

### 2.7. Attention Controller Module

In our model, the attention module prevents idle behaviour from disrupting more deliberate and at times, immediate reactive and communicative behaviour. Idle behaviour must be paused if an event that requires immediate attention occurs. There are two types of events that require immediate attention: Sudden environment changes, and events pertaining to the interaction. For example, if the user starts to wave at the agent, the agent cannot gaze idly or avert their head. Conversely, during the rock-paper-scissors game, it does not make sense if the agent averts their gaze when playing a hand. If a behaviour command for a specific joint of the agent enters the animation engine right after another command for the same joint, the resulting movement will blend both animations. For example, if the agent is showing the “rock” hand gesture and a scratching head gesture comes after that, the rock gesture blends with the head scratch. This is not desirable. The attention module is designed to avoid such unwanted blending. The Attention Controller module attempts to balance the realism of blended natural, ongoing, complex behaviour with more deliberate reactive behaviour. The scope of the Attention Controller can be gaze, body, or both. If the attention signal only requires the attentiveness of the gaze, other body parts can continue with their idle or ongoing behaviour. The same rule applies to the body. Similar to Sidner and colleagues' work, the design leads to three phases for attention: establishing, maintaining, and ending attention (Sidner et al., 2004). When attention-seeking events occur, establishing the attention occurs by triggering a flag for gaze, body or both. During the maintaining phase, the FSM time-based attention flag continues for a fixed period of time and then turns off automatically (closing) unless another attention request is received. In the maintaining phase, depending on whether it is the gaze or body attention flag that is in the on position, their corresponding idle behaviour pauses until the flag turns off.

### 2.8. Behaviour Scheduler Module

In our system, several modules generate behaviour requests. While some of these requests can be realised in parallel (e.g., head avert to a point and scratching the neck), others are mutually exclusive because they compete for the same actuators (e.g., waving for the user and scratching the chest). Sending two behaviour requests which share some of the joints to the animation engine can lead to ignoring one of the behavioural acts or blending the acts in an undesired way. This can be problematic, especially for behaviour that is time-based and crucial. Therefore, there is a need for a mechanism for prioritising and selecting behaviour.

To control overlapping behaviours, the system uses two mechanisms: priority queues and scheduling. Any behaviour that the model generates is assigned to either the high, mid, or low priority queue. High-priority behavioural acts need to be performed immediately after the generation. They are usually a reaction to the environment, user inputs, or driven by the interaction scenario requiring an immediate response. For mid-priority behaviour, even if the behaviour cannot be scheduled for the specific time, it still needs to be scheduled as close as possible. The low-priority behaviour is usually idle behaviour that does not correspond in a synced way to any outside events so that their delay or removal will not affect the perceived experience. Behaviour is inserted into the corresponding queues in multiple behaviour controller modules. The Behaviour Scheduler then sends the selected behaviour (with the highest priority) to the animation engine. The scheduling mechanism is responsible for deciding which behaviour is selected. If two behavioural acts have different priorities, the one with a higher priority will be selected. If a higher priority task is followed by a lower priority task, the attention module will assure that the lower priority task does not affect or blend with the higher priority behaviour. If a low priority behaviour is followed by a higher priority or low priority behaviour, it will blend with the new behaviour.

### 2.9. Implementation of the Real-Time Interactive System

This section describes the implementation of the model to control a virtual human in real-time. The graphical programming environment MathWorks “Simulink” (http://mathworks.com/products/simulink/) is used to implement the model itself. Simulink is a well-established environment for continuous and discrete domain simulations. Key advantages are the graphical system construction, support for real-time simulations, and the availability of data visualisation tools (Bernardet et al., 2016a). Importantly, Simulink natively integrates a finite state machine component “Stateflow” (http://mathworks.com/products/stateflow) which allows constructing hybrid control systems within a single application (Sahbani and Pascal, 2000).

**Figure 6:**
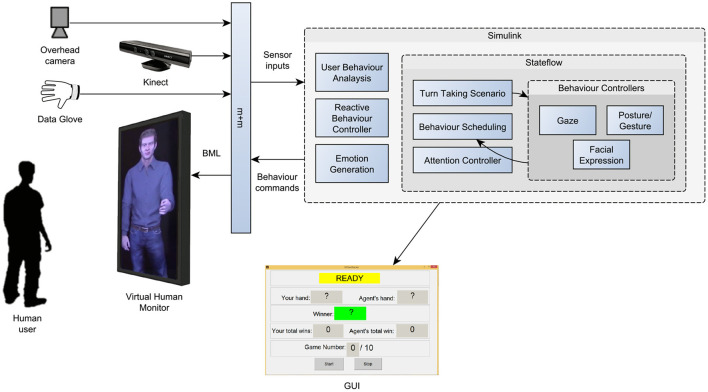
Implementation of the model as a real-time interactive system. The continuous control model is implemented using Simulink. The Stateflow component realises the finite state machine for event-based control. The model receives sensor data and sends control signals to the Virtual Human using the “Movement + Meaning” (m+m) middleware (Bernardet et al., 2016b).

The inputs of the system are continuously received from sensors installed in the environment (Figure 6). These sensors include a Microsoft Kinect 3D camera (https://developer.microsoft.com/enus/windows/kinect/develop) for gesture recognition and an overhead camera to track the users' location in space. A custom-made glove was used to sense the bending of the users' thumb, index, and middle finger. The outputs of the system are facial expressions, postures, and gestures of the 3D virtual human. The “Smartbody” animation toolkit is used as the engine to animate the Virtual Human (Shapiro, 2011). While the Behaviour Markup Language (BML) is used to send commands to the Smartbody agent (Kopp et al., 2006), the Movement + Meaning (m+m) middleware acts as a communication's interface between different components (Bernardet et al., 2016b).

## 3. Experimental Evaluation

To assess if and how well the predicted model parameters convey a specific personality category, we conducted two types of experiments: In the passive, video-based experiments, participants rated clips of the idle behaviour of a virtual character captured from running the simulation setting the parameters such as to generate distinct personality categories. In the presential experiment, participants interacted with the virtual character by playing a game of Rock-Paper-Scissors and subsequently rated the agent's personality. No participants took part in both experiments.

### 3.1. Passive Test of the Effectiveness to Convey Personality

**Figure 7:**
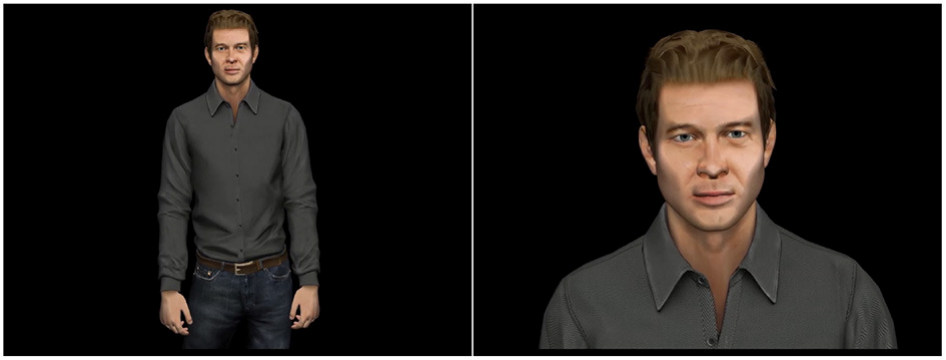
Still image of full-body (left) and head-only (right) video used in the passive experiment.

To determine how the overall available information affects the impression of personality, we ran a study that compared head-only and full-body videos of the virtual human. For the head-only condition, the video was zoomed to the face of the character, while in the full-body video, the face was neutral and did not have any facial expression (Figure 7). Independent variables of the experiment were the framing of the video and the personality category intended to be perceived by the user. Five categories, corresponding to the combination of the extremes of the two dimensions of Extraversion and emotional stability plus a centre point were tested: High Extraversion/Low Stability (HELS), High Extraversion/High Stability (HEHS), Low Extraversion/Low Stability (LELS), Low Extraversion/High Stability (LEHS), Mid Extraversion/Medium Stability (Neutral). Dependent variables are the perceived level of extraversion and emotional stability of the virtual human.

#### 3.1.1. Participants

A total of 23 graduate and undergraduate students of the Simon Fraser University and the University of British Columbia, aged between 22 and 45 years old, participated in the study in return for course credit. Of the participants, 15 identified as female and 8 as male.

#### 3.1.2. Personality Inventory

The Ten-Item Personality Inventory (TIPI) (Gosling et al., 2003) was used to assess the “Big Five” personality traits of openness, conscientiousness, extraversion, agreeableness and emotional stability. The TIPI scale is fast to administer, reliable and has been shown to yield a strong positive correlation between raters and self-reported personality (Gosling et al., 2003).

#### 3.1.3. Procedure

Participants first read an instruction page that described the process. Next, they were presented with fourteen 30 s videos of a physically identical male virtual character showing different personality categories through idle behaviour such as head movement, weight-shift and scratching the body parts. The videos were created as every combination of gesture extraversion level (High, Low), gesture emotional stability level (High, Low), and framing (head-only, full-body) yielding 8 total clips (2 x 2 x 2), in addition to two neutral clips for use in the experiment. The length of 30 s was chosen based on experimental evidence that shows this time is long enough for people to form an impression about the personality (Borkenau et al., 2004; Carney et al., 2007). The videos were presented in random order. After each video, participants were asked to assess the personality of the agent using the TIPI. Additionally, they were given one question about the dominance of the character. The participants were not allowed to replay the videos and return to previous videos. At the end of the experiment, participants were asked a general question about the experiment. The experiment ended with a TIPI in which participants rated the extent to which the pair of traits applied to themselves. The experiment took, on average, approximately 30 min to complete. The experiment was implemented using the jsPsych library (de Leeuw, 2015). and ran on a server on the Simon Fraser University, Surrey campus.

### 3.2. Real-Time Interactive Evaluation

During the presential experiment, there is a need for a scenario of the interaction between the virtual character and participants, and the virtual character needs to be responsive to the environmental inputs. During the presential experiment participants dynamically interacted with the virtual character which behavioural acts were continuously controlled by the system. The system is designed in a way that it attempts to generate proper behaviour for the character to maintain a natural interaction with users. One of the steps along this line has been to develop some scenarios that allow users to interact with the character. During the interaction following the storey of the scenario, we can see if the character sustains an interaction with a user and how users assess the experience. The designed test case scenario reduces the problem set while developing and testing the system. Additionally, an interactive environment affords the induction of a stronger impression of personality traits. Our goal was to create an easy-to-learn and engaging scenario that provides an interactive environment with no speech. The reason to avoid the speech was to concentrate only on the nonverbal behaviour of the character.

#### 3.2.1. The Rock-Paper-Scissors Game Scenario

**Figure 8:**
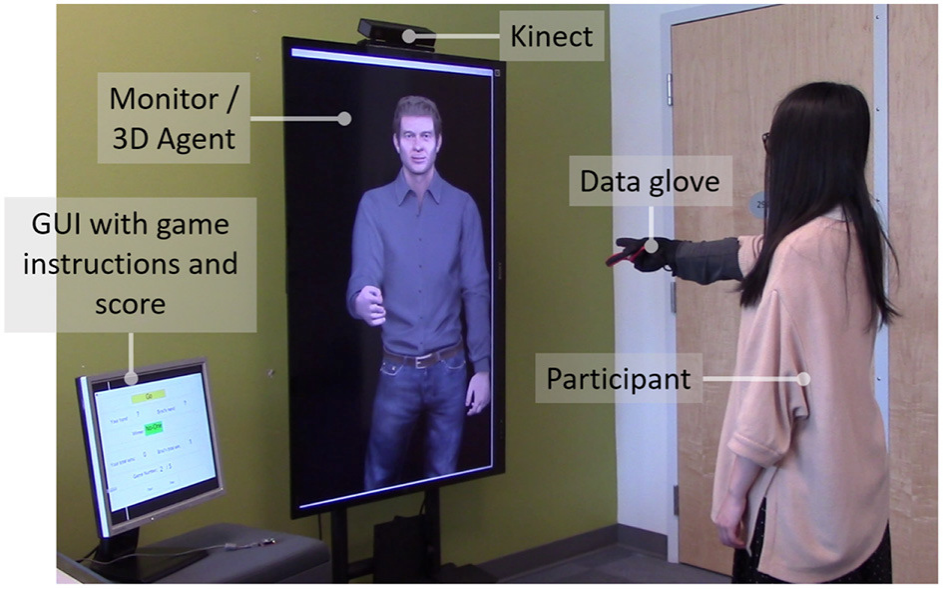
Participant interacting with the virtual human during the Rock-Paper-Scissors game for the interactive evaluation.

The Rock-Paper-Scissors (RPS) game scenario was designed to provide a framework for the interaction between a user and a virtual human in real-time with no need for verbal exchange. During the game, the virtual character plays multiple rounds of the rock-paper-scissors game with the user (Figure 8). A Graphical User Interface (GUI) displayed on a separate smaller monitor within view of the user is used for synchronising the Rock-Paper-Scissors game. The GUI is updated based on the states of the interaction (Ready, Go, Hands, Result). The game starts with GUI announcing and demonstrating “Ready…Go.” In the “Go” state, a random hand gesture is generated for the character. Then, the 3D virtual character and the participant both simultaneously showed a rock, paper or scissor hand gesture while the GUI was updated. The character's choice of hand gesture is randomly chosen from the three possibilities. The winner of the game and total wins so far were calculated and updated in GUI. The interactive experiment used the same five intended personalty categories: High Extraversion/Low Stability (HELS), High Extraversion/High Stability (HEHS), Low Extraversion/Low Stability (LELS), Low Extraversion/High Stability (LEHS), Mid Extraversion/Medium Stability (Neutral).

#### 3.2.2. Participants

A total of 41 graduate and undergraduate students of the Simon Fraser University and the University of British Columbia participated in the experiment in return for extra course credit. The participants were between 22 and 45 years old. Of the participants, 25 identified as female and 16 as male.

#### 3.2.3. Procedure

The experiment started by asking participants to review and sign the consent form. Then, they answered the TIPI questions to rate their own personality. Next, they put on the data glove and did a practise round of the rock-paper-scissors game with the character, during which the process and the designed graphical user interface (GUI) were explained. The participant's hand gestures were captured using a custom-made data glove developed in our lab, specifically for the system. The glove consisted of three bend sensors which measured if the hand's fingers are bent. The participants stood in front of a 60-inch TV monitor showing an approximately life-sized virtual character. After performing a practice round, participants started with the experiment. The experiment consisted of five sessions of the Rock-Paper-Scissors game for five different personality categories. In the five sessions, a combination of four different personality categories and one neutral behaviour were applied to the character. In each of these sessions, five rounds of rock-paper-scissors game were played to give participants enough time to form an impression about the agent's personality. Each session of the game took approximately 2 min. The order of the personality categories was randomised. The experiment concluded with a short semi-structured interview asking participants about their general experience. To assess the impression of personality, we used a paper version of the Ten-Item Personality Inventory (TIPI).

## 4. Results

### 4.1. Conveying the Intended Personality Dimensions

A key hypothesis of our work is that personality is expressed through a specific combination and characteristic of non-verbal behaviour, specifically in the interaction between two persons. Subsequently, we will refer to the intended personality attribution as the “Target.” To test this hypothesis, we conducted a one-way within-subject MANOVA of the dependent variables of attributed level of Extraversion and Stability for the *interactive* experiment.

**Figure 9:**
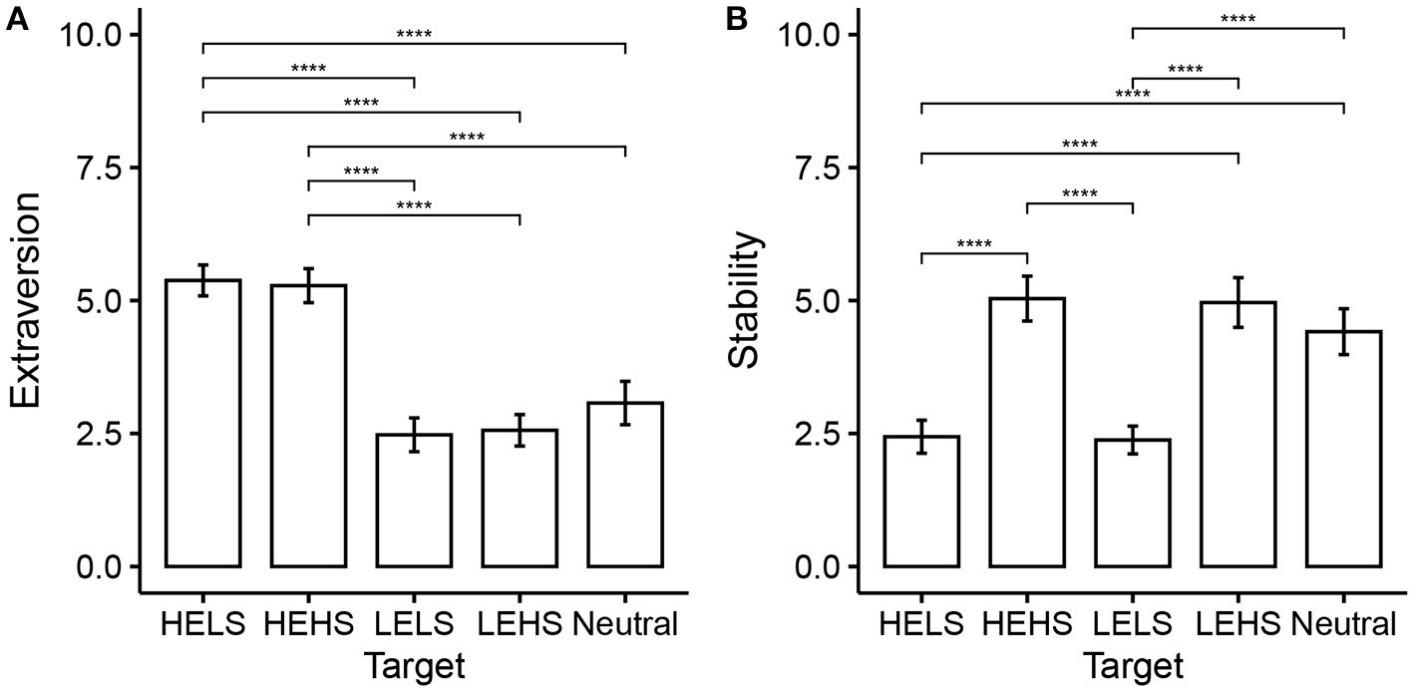
Comparison of interaction mode. Error bars indicate 95% confidence interval. Extraversion **(A)** and Stability **(B)**.

The analysis showed a significant effect of TargetPersonality [*F*_(8, 318)_ = 67.78, *p* < 0.001, Wilk's λ = 0.14]. The subsequent univariate analysis is conducted for Extraversion and Stability separately. The results of a one-way within ANOVA (Figure 9A) show that there is a significant effect of ‘Target Extraversion' on the Extraversion rating [*F*_(1, 40)_ = 2462.98, *p* < 0.001, partial η^2^ = 0.98, *F*_(4, 160)_ = 81.72, *p* < 0.001, partial η^2^ = 0.67. No sphericity correction applied; all *p*-values for Mauchly's test *p* > 0.05]. Importantly, the *post-hoc* pairwise analysis with Bonferroni correction shows that Neutral (*M* = 3.07, *SD* = 1.29) was significantly different from both HEHS (*M* = 5.28, *SD* = 1.01) [*t*_(40)_ = 8.47, *p* < 0.001, *d* = 1.32] and HELS (*M* = 5.38, *SD* = 0.92] [*t*_(40)_ = 9.53, *p* < 0.001, *d* = 1.49]. No significant differences were found between Neural and either LEHS (*M* = 2.56, *SD* = 0.94) [*t*_(40)_ = –2.07, *p* = 0.453, *d* = –0.32) or LELS (*M* = 2.48, *SD* = 1.01) [*t*_(40)_ = –2.19, *p* = 0.341, *d* = –0.34]. The midpoint of the Ten Item Personality Measure is at 4. Hence the Neutral condition with *M* = 3.07 was rated lower in Extraversion than intended.

In the rating for “Stability” (Figure 9B) we found a significant effect of “Target Stability” on the Stability rating [*F*_(1, 40)_ = 1337.38, *p* < 0.001, partial η^2^ = 0.97, *F*_(4, 160)_ = 55.87, *p* < 0.001, partial η^2^ = 0.58. No sphericity correction applied; all *p*-values for Mauchly's test *p* > 0.05]. The *post-hoc* pairwise analysis with Bonferroni correction shows that Neutral (*M* = 4.41, *SD* = 1.36) was significantly different from both HELS (*M* = 2.44, *SD* = 0.98) [*t*_(40)_ = –7.54, *p* < 0.001, *d* = –1.18] and LELS (*M* = 2.38, *SD* = 0.83) [*t*_(40)_ = –8.22, *p* < 0.001, *d* = –1.28]. No significant differences were found between Neural and either HEHS (*M* = 5.04, *SD* = 1.34) [*t*_(40)_ = 2.23, *p* = 0.316, *d* = 0.35] or LEHS (*M* = 4.96, *SD* = 1.48) [*t*_(40)_ = 2.07, *p* = 0.446, *d* = 0.32].

### 4.2. How Was the Personality Conveyed?

#### 4.2.1. Comparing the Mode of Interaction

**Figure 10:**
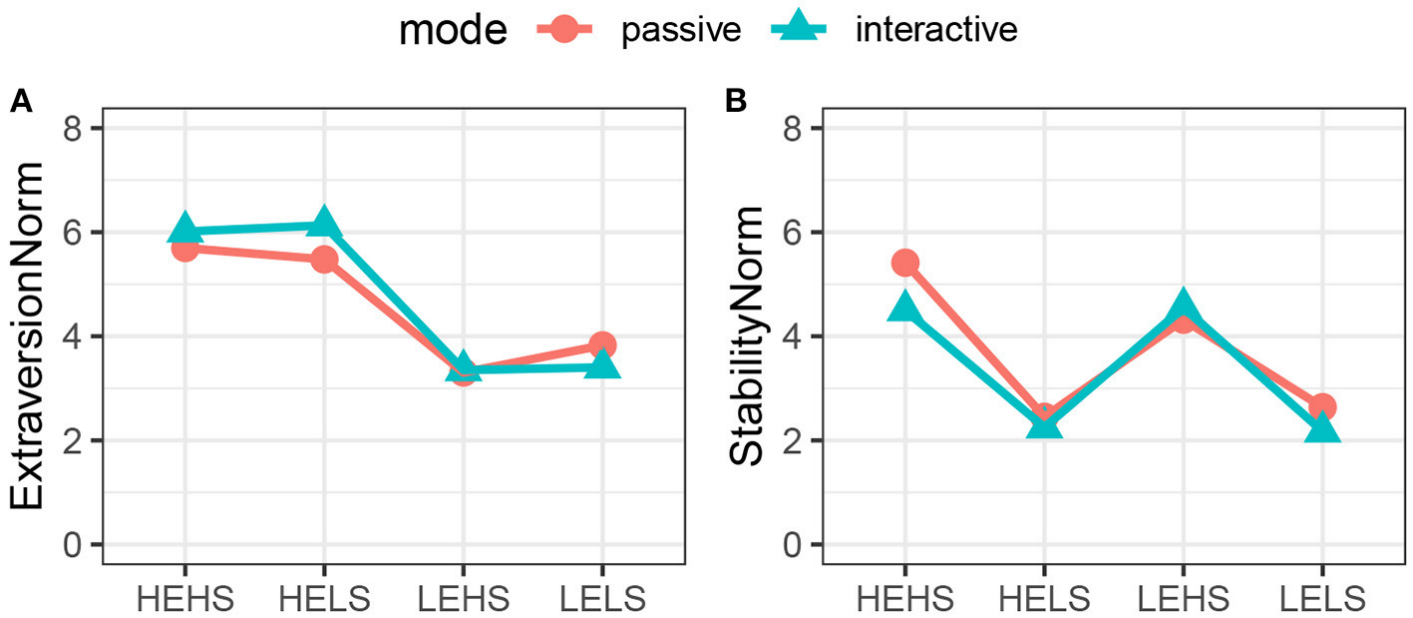
Comparing the passive with the interactive mode of interaction. Extraversion **(A)** and Stability **(B)**.

To assess the effectiveness of the model to convey a target personality impression, we had tested both, a passive condition where participants watched a video and an active condition in which participants interacted with the agent. Next, we will assess the effect of the mode of interaction on Extraversion and Stability. In order to do so, we normalised the ratings using the neutral condition and combine the data from the passive and active experiments. This allows us to run a two-way, mixed-mode MANOVA with the factors of Target Personality and Mode. The MANOVA showed a significant main effect for Target Personality [*F*_(6, 370)_ = 129.05, *p* < 0.001, Wilk's λ = 0.1] and a significant interaction effect between Target Personality and mode of interaction [*F*_(6, 370)_ = 2.58, *p* < 0.05, Wilk's λ = 0.92]. However, no main effect for mode of interaction [*F*_(2, 61)_ = 0.57, *p* > 0.05, Wilk's λ = 0.98] was found. The subsequent two-way ANOVA for Extraversion (Figure 10A) showed no significant interaction between the mode of interaction and Target Personality [*F*_(3, 186)_ = 3.50, *p* = 0.017, partial η^2^ = 0.05. No sphericity correction applied; all *p*-values for Mauchly's test *p* > 0.05]. We found a significant main effect for Target Personality [*F*_(3, 186)_ = 120.08, *p* < 0.001, partial η^2^ = 0.66], but not for mode of interaction [*F*_(1, 62)_ = 0.44, *p* = 0.508, partial η^2^ < 0.01]. The results of the two-way ANOVA for Stability (Figure 10B) replicate the above findings: No significant main effect for mode of interaction [*F*_(1, 62)_ = 0.91, *p* = 0.344, η^2^ = 0.01] or interaction [*F*_(2.61, 161.57)_ = 1.72, *p* = 0.173, η^2^ = 0.03] between the factors, and a significant main effect for Target Personality [*F*_(2.61, 161.57)_ = 110.91, *p* < 0.001, η^2^ = 0.64]. Sphericity corrections: The effect for Stability (Mauchly's *W* = 0.78, *p* = 0.011) and mode*Stability (Mauchly's *W* = 0.78, *p* = 0.011) were adjusted using the Greenhouse-Geisser correction).

#### 4.2.2. Effect of the Available Visual Information

**Figure 11:**
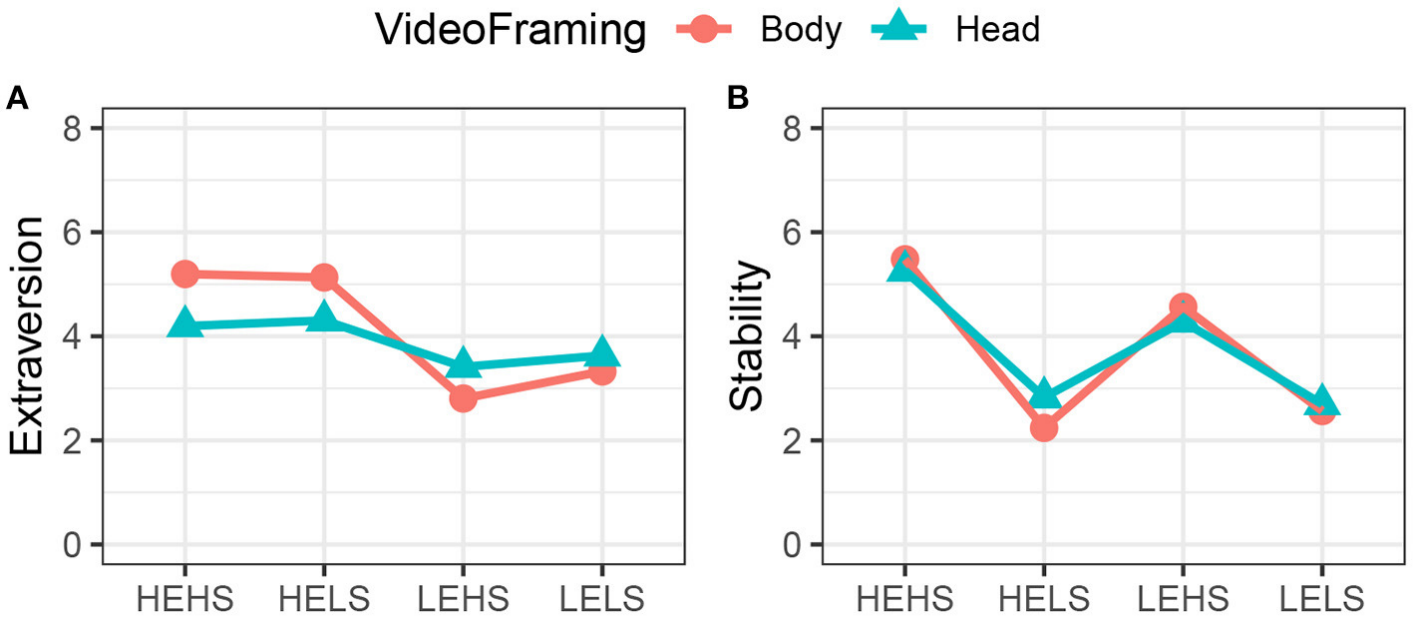
Comparison of video framing (head-only vs. full-body) in the passive experiment. Extraversion **(A)** and Stability **(B)**.

In the passive experiment, we manipulated the amount of available visual information by showing the participants videos that were framed full-body or head-only. This provides some indication as to what channels are used to convey the personality impression (Figure 11). As previously, we normalised the ratings using the neutral condition. Note that we only include the full-body data from the passive experiment.

A two-way mixed-mode MANOVA did not show a significant main effect for VideoFraming [*F*_(2, 87)_ = 1.62, *p* > 0.05, Wilk's λ = 0.96], but a significant main effect for Target Personality [*F*_(6, 130)_ = 29.21, *p* < 0.001, Wilk's λ = 0.18] as well as a significant interaction effect [*F*_(6, 174)_ = 5.4, *p* < 0.001, Wilk's λ = 0.71]. A a subsequent two-way, within-subject ANOVA for Extraversion yields a significant interaction effect between VideoFraming and Target Personality [*F*_(3, 66)_ = 8.22, *p* < 0.001, η2 = 0.27. No sphericity correction applied; all *p*-values for Mauchly's test *p* > 0.05]. As can be seen in Figure 11A, the availability of the full-body information increases the ratings for HEHS/HELS, while lowering those for LEHS/LELS.

The ANOVA for Stability shows that the main effect for Target Personality is significant [*F*_(1.95, 42.90)_ = 47.21, *p* < 0.001, η2 = 0.68]. Sphericity corrections: The effect for Stability (Mauchly's *W* = 0.44, *p* = 0.004) was adjusted using the Greenhouse-Geisser correction). Interestingly, for Stability, we find neither a significant interaction effect [*F*_(3, 66)_ = 1.77, *p* = 0.162, η^2^ = 0.07] nor a significant main effect of VideoFraming [*F*_(1, 22)_ = 0.14, *p* = 0.709, η^2^ < 0.01]. Hence, the framing does not affect the perception of Stability as much as it does the one for Extraversion (Figure 11B).

## 5. Discussion and Conclusion

In this paper, we present the “RealAct” Model that proposes a mechanistic hypothesis of the aetiology of the two big five dimensions of extraversion and emotional stability. Uniquely, the comprehensive model affords a real-time interaction between the model embodied in a virtual humanoid agent and a participant. The model comprises the following six elements:

1. A gaze controller that refines the “eyes alive” model of gaze (Lee et al., 2002) to create a gaze behaviour following the human ocular behaviour,
2. A posture and gesture controller that conveys the impression of personality through the expressiveness posture-shifts, leaning, self-adaptors, body twitches and spacial gestures,
3. A facial expression controller where the impression of personality is created through changing the intensity of emotions expression and filtering, as well as facial twitches,
4. An emotion generation module that integrates valence and arousal when emotional triggers are activated. In this module, personality affects the type and impact of the interaction and environment triggers,
5. An attention controller that makes the 3D character attentive to events,
6. A behaviour scheduler that prioritises and selects a behaviour with the highest priority from multiple behaviour requests using three priority queues for high, mid and low priority behaviour.

In our experimental studies, we assessed three aspects of our model. Firstly, we assessed the amounts of extraversion and emotional stability that participants attribute to a character depending on a specific combination and mode of execution of behaviours (gaze, facial expressions, gestures, and postures). Secondly, we investigated how the amount of visual information—formalised as the framing of the video—affects the impression of personality. Thirdly, we tested the hypothesis that the real-time interaction between a human and a virtual character should strengthen the impression of extraversion and enhance the perception of emotional stability for the virtual character.

The experiments showed that non-verbal behaviour of the agent conveys the impression of distinct personality dimensions of extraversion and stability; the amount of extraversion and emotional stability that participants attributed to the virtual human depended on a specified combination of facial expression, eye gaze, body posture, and gestures that the agent exhibited. In particular, characters showing fast and spacious gestures, frequent and long mutual gazes, and frequent intensive positive emotions were judged as extravert while slow movements, short duration of mutual gaze and frequent and long periods of averting gaze and not strong emotions (philtre) were correlated with introverts. Additionally, characters showing frequent scratches, twitches, blinks, and frequent and strong negative emotions were judged as emotionally unstable where lack of twitch and scratch and lack of expression of strong negative emotions were associated with emotional stability. Compared to the neutral condition, the model was able to increase the amount of attributed extraversion, but not decrease it. We observed the opposite effect for stability; decreasing the amount of stability worked, but not increasing it did not. This is intuitively understandable; more extraversion and less stability are both correlated with an increase in the amount of activation.

The results from our experiment that compared the different amount of visual information available indicate that, while facial expression plays a major role, seeing the entire body gives a stronger impression for extraversion. The absence of a strong effect of the interaction modes—passive videos vs. presential real-time interaction—was somewhat surprising. However, the real-time interaction presents a specific challenge, in that the system has to be able to maintain a consistent and plausible interaction. We see it as a significant achievement that the model was able to meet this additional difficulty and that participants did not experience a break in presence (Slater and Steed, 2000), e.g., because of the agent behaving unrealistically or erratically. Not least, this ability of the model also lends support to the control theoretical approach that combines continuous with discrete control and derives its parameters from empirical studies. We believe that systems theory indeed provides an adequate level of abstraction and a theoretical framework that will eventually allow closing the gap between black-box models and low-level descriptions at the neuronal level (Bischof, 1998).

### 5.1. Current Limitations and Future Work

In our evaluation study, all experiments were conducted using the same White male character. This has helped to reduce variability but also limits generalisability. In future experiments, we plan to use a range of characters reflecting different cultural, ethnic, gender, and age backgrounds. This would allow finding the generic mechanisms that are universal across age, gender, ethnicity etc. The ability to do this relatively easily with virtual humans is in fact an important advantage of this approach, though it incurs the disadvantage that the sample size would need to be much larger.

The biggest caveat of the current study is that the behaviour of the agent was not recorded at the ‘motor level”, i.e., the exact time course of each join. Doing so would allow a direct comparison with motion captured data from human models, and would allow closing the circle by making sure that the intended modulation of behaviour execution was actually effected.

The setup used in our study—live interaction via a 2D screen—is akin to a video-conferencing system, rather than interaction in person. The rationale for this choice was two-fold; on the one hand, humans have no problem interacting with others and making judgements about them in mediated interaction, and on the other hand, the vast majority of research on how personality is attributed based on observed behaviour uses pre-recorded, 2D videos as stimuli. To our knowledge, there exist no studies that systematically compare the effect of meditated and live interaction. Hence, replicating our study using immersive Virtual Reality could help better understand the contribution of the interaction medium. The Rock-Paper-Scissors scenario has worked well to develop and test the model, further interaction scenarios are needed to expand the scope of the model. Suitable scenarios are likely to also come from the application of virtual humans in domains such as education and health (Dar and Bernardet, 2020).

Though we used a large set of movement parameters for all channels of behaviour, there are still many factors such as fluidity and smoothness of gestures that merit further investigation. We have shown that behaviour control parameters are highly correlated with the users' impression of personality. However, a more detailed analysis of the specific kinematic movement properties that led to the attribution of the personality categories would provide valuable insights. Our model presently provides a mechanistic explanation of two of the Big Five personality dimensions: Extraversion and Neuroticism. The inclusion of the personality dimensions of Openness, Agreeableness, and Conscientiousness is a natural extension of our model. However, these dimensions are more “cognitive” and hence would transcend what can be expressed using non-verbal behaviour. They would likely need verbal communication or at least interaction that is more transactional such as negotiation (DeVault et al., 2015). There is a clear role for machine learning in agent behaviour control models, e.g., for associating gestures with speech, such as our work speech accompanying gestures based on autoregressive neural network (Nagy et al., 2021). However, employing machine learning to real-time interaction control as a whole is a much more challenging feat due to the inherent open-endedness and therefore lack of training data. This is compounded by the unsolved question of what the epistemological value is of unstructured machine learning models since their very complexity easily becomes untrackable and a barrier to understanding itself (Frické, 2015; Carabantes, 2019).

## Data Availability Statement

The raw data supporting the conclusions of this article will be made available by the authors, without undue reservation.

## Ethics Statement

The studies involving human participants were reviewed and approved by Office of Research Ethics, Simon Fraser University, Vancouver, Canada. The patients/participants provided their written informed consent to participate in this study.

## Author Contributions

MS model development and implementation, experiment implementation, data collection, data analysis, and original manuscript text. SD model development, supervision, and manuscript editing. UB personality modelling, implementation interactive system, data analysis, and manuscript writing. All authors contributed to the article and approved the submitted version.

## Conflict of Interest

The authors declare that the research was conducted in the absence of any commercial or financial relationships that could be construed as a potential conflict of interest.

## Publisher's Note

All claims expressed in this article are solely those of the authors and do not necessarily represent those of their affiliated organizations, or those of the publisher, the editors and the reviewers. Any product that may be evaluated in this article, or claim that may be made by its manufacturer, is not guaranteed or endorsed by the publisher.
